# Integrating molecular subtype and CD8^+^ T cells infiltration to predict treatment response and survival in muscle-invasive bladder cancer

**DOI:** 10.1007/s00262-024-03651-3

**Published:** 2024-03-02

**Authors:** Bingyu Li, Kaifeng Jin, Zhaopei Liu, Xiaohe Su, Ziyue Xu, Ge Liu, Jingtong Xu, Hailong Liu, Yuan Chang, Yiwei Wang, Yu Zhu, Zewei Wang, Le Xu, Weijuan Zhang

**Affiliations:** 1 Department of Immunology, School of Basic Medical Sciences, Fudan University, Shanghai, China; 2 NHC Key Laboratory of Glycoconjugate Research, Department of Biochemistry and Molecular Biology, School of Basic Medical Sciences, Fudan University, Shanghai, China; 3 Department of Urology, Zhongshan Hospital, Fudan University, Shanghai, China; 4 Department of Urology, Fudan University Shanghai Cancer Center, Shanghai, China; 5 Department of Urology, Xinhua Hospital, Shanghai Jiao Tong University School of Medicine, Shanghai, China; 6 Department of Urology, Shanghai Ninth People’s Hospital, Shanghai Jiao Tong University School of Medicine, Shanghai, China; 7 Department of Urology, Ruijin Hospital, Shanghai Jiao Tong University School of Medicine, Shanghai, China

**Keywords:** Muscle-invasive bladder cancer, Molecular subtype, CD8^+^ T cells infiltration, Chemotherapy, Immunotherapy

## Abstract

**Background:**

Luminal and Basal are the primary intrinsic subtypes of muscle-invasive bladder cancer (MIBC). The presence of CD8^+^ T cells infiltration holds significant immunological relevance, potentially influencing the efficacy of antitumor responses. This study aims to synergize the influence of molecular subtypes and CD8^+^ T cells infiltration in MIBC.

**Methods:**

This study included 889 patients with MIBC from Zhongshan Hospital, The Cancer Genome Atlas, IMvigor210 and NCT03179943 cohorts. We classified the patients into four distinct groups, based on the interplay of molecular subtypes and CD8^+^ T cells and probed into the clinical implications of these subgroups in MIBC.

**Results:**

Among patients with Luminal-CD8^+^T^high^ tumors, the confluence of elevated tumor mutational burden and PD-L1 expression correlated with a heightened potential for positive responses to immunotherapy. In contrast, patients featured by Luminal-CD8^+^T^low^ displayed a proclivity for deriving clinical advantages from innovative targeted interventions. The Basal-CD8^+^T^low^ subgroup exhibited the least favorable three-year overall survival outcome, whereas their Basal-CD8^+^T^high^ counterparts exhibited a heightened responsiveness to chemotherapy.

**Conclusions:**

We emphasized the significant role of immune-molecular subtypes in shaping therapeutic approaches for MIBC. This insight establishes a foundation to refine the process of selecting subtype-specific treatments, thereby advancing personalized interventions for patients.

**Supplementary Information:**

The online version contains supplementary material available at 10.1007/s00262-024-03651-3.

## Introduction

Bladder cancer is one of the most common genitourinary malignancies with a high mortality risk [1]. Within this context, approximately a quarter of cases are identified as muscle-invasive bladder cancer (MIBC), carrying an unfavorable prognosis [2]. Over the past decades, platinum-based chemotherapy has been regarded as the standard care for MIBC patients [3]. For those patients ineligible or resistant to platinum-based treatment, certain immune checkpoint inhibitors have gained clinical benefit and have been approved for MIBC treatment in recent years. Despite this, the favorable therapeutic outcomes have remained confined to a limited patient subset [4]. This therapeutic landscape has facilitated the development of a more precise, biology-driven classification of MIBC, aimed at enhancing clinical management for patients.

The molecular classification of bladder cancer has emerged as a pivotal approach to understanding the diversity of MIBC. Previous studies shown that two primary intrinsic subtypes of MIBC have emerged—Luminal and Basal [5–7]. These intrinsic subtypes hold immediate potential in guiding both prognosis and treatment strategies for MIBC. Notably, it has come to light that the MIBC with the basal feature is linked to reduced disease-free survival (DFS) and overall survival (OS) in comparison with the luminal subtypes [6]. Furthermore, a significant proportion of Basal MIBC cases could get benefit from chemotherapy, contrasting with the resistance observed in Luminal subtype tumors [6]. However, the responses to immune therapies were observed not only in metastatic urothelial carcinoma (mUC) patients with luminal features but also in those with basal features [8].

Beyond the genetic and molecular attributes, the infiltration and functional status of immune cells exert a considerable influence on patient prognosis and treatment responses. Notably, the CD8^+^ T cells have emerged as a pivotal immune parameter with the potential to decisively impact effective antitumor responses [9–11]. Numerous researches have demonstrated the correlation between CD8^+^ T cells infiltration and improved prognosis as well as enhanced therapeutic effectiveness [12, 13]. Our previous investigations also emphasize the significant heterogeneity of intratumoral CD8^+^ T cells in the context of MIBC. These intratumoral CD8^+^ T cells exhibit diverse phenotypes that ultimately influence the direction of the anti-tumor immune response [14–16].

In this study, we established an immunomolecular subtype classification, achieved by integrating and categorizing molecular subtypes alongside CD8^+^ T cells infiltration into four distinct groups within MIBC: Luminal-CD8^+^T^high^, Luminal-CD8^+^T^low^, Basal-CD8^+^T^high^ and Basal-CD8^+^T^low^. Subsequently, we conducted an in-depth analysis encompassing clinicopathological, genomic, and immunophenotypic attributes across these four stratified subgroups. Moreover, we investigated how this novel classification influences both chemotherapy and PD-L1 blockade therapy outcomes in patients with MIBC.

## Materials and methods

### Study cohorts

The study utilized data from four independent patient cohorts, consisting of a total of 889 patients (Supplementary Fig. 1A). For our local Zhongshan Hospital Affiliated to Fudan University (ZSHS) cohort, with the approval of the Clinical Research Ethics Committee of Zhongshan Hospital affiliated to Fudan University, 142 patients who were diagnosed with MIBC and underwent radical cystectomy at Zhongshan Hospital from 2002 to 2014 were followed up regularly till July 2016. Among them, 7 patients were excluded from the analysis due to dot loss on tissue microarray (TMA). Of these, 64 patients received adjuvant chemotherapy to reduce the risk of disease relapse according to the guidelines and the preferences of the patients, while 71 did not receive any neoadjuvant or adjuvant therapy. In clinical settings, platinum-based chemotherapy remains the standard for treating advanced and metastatic bladder cancer [17]. Our local ZSHS cohort maintained consistency in systemic therapy over a 12-year period, with patients being administered platinum-based chemotherapy rather than immunotherapy. The Cancer Genome Atlas (TCGA) cohort encompasses 391 MIBC patients with comprehensive clinical profiles. Initially, a pool of 412 patients diagnosed with bladder cancer was sourced from the TCGA network using TCGA-Assembler 2.0.6 as of July 2021. Subsequently, 21 cases were excluded from this pool due to reasons such as non-muscle-invasive bladder cancer (NMIBC) pathologic diagnosis (*n* = 4), absence of survival or mRNA data (*n* = 7), or prior neoadjuvant therapy (*n* = 10). Regarding the IMvigor210 cohort, a phase II clinical trial that comprises 348 individuals diagnosed with mUC treated with the PD-L1 inhibitor atezolizumab, clinical and RNA-seq data were procured from http: //research-pub.gene.com/IMvigor210CoreBiologies utilizing the "IMvigor210CoreBiologies" R package. As for the phase II clinical trial NCT03179943 cohort, it encompassed 21 patients who underwent treatment with guadecitabine and atezolizumab. Ultimately, the mRNA data of 15 patients were incorporated into this study. Detailed clinical and pathological attributes are presented in Supplementary Tables 1–3.

### Processing of genomic and transcriptomic data

For transcriptomic analyses, RNA-seq data were normalized through the formula Log_2_(FPKM + 1). Immune cells infiltration signature including CD8^+^ T cells signature [18], effector CD8^+^T signature [4], exhausted CD8^+^T signature [19] and immune function signature including IFN-γ-related signature [20], pan fibroblast TGF-β signature [21] were calculated by Single sample gene set enrichment analysis (ssGSEA) algorithms based on related genes expression as previously reported (genes are listed in Supplementary Table 4). ssGSEA algorithms was accomplished by a ‘single sample’ extension of GSEA that allows one to define an enrichment score that represents the degree of absolute enrichment of a gene set in each sample within a given data set. The gene expression values for a given sample were rank-normalized, and an enrichment score was produced using the empirical cumulative distribution functions (ECDF) of the genes in the signature and the remaining genes [22].

For genomic analyses, tumor mutation burden (TMB) was defined as total non-silent somatic mutation per megabase (mut/Mb) which was acquired from https://portal.gdc.cancer.gov/. The copy number variants (CNV) data in TCGA cohort originated from http://www.cbioportal.org. Amplified or deleted regions of the genome were identified using the Genomic Identification of Significant Targets in Cancer (GISTIC) 2.0 algorithm, which divided into five discrete steps: (1) accurately defining the copy-number profile of each cancer sample; (2) identifying the somatic copy number alterations (SCNAs) that most likely gave rise to these overall profiles and estimating their background rates of formation; (3) scoring the SCNAs in each region according to their likelihood of occurring by chance; (4) defining the independent genomic regions undergoing statistically significant levels of SCNA; and (5) identifying the likely gene target(s) of each significantly altered region [23].

### Immunohistochemistry and assay method

Serial tissue sections fixed in formalin and embedded in paraffin were used to construct TMA and the antibodies used for immunohistochemistry (IHC) staining are summarized in Supplementary Table 5. IHC staining for CD8^+^ T cells and other molecules were carried out according to previous protocols [14, 24, 25] (representative IHC images, Supplementary Fig. 1B). As before, the infiltration density of immune cells was evaluated as the mean value of cells/HPF under 3 randomized high-power magnification filed (HPF, × 200 magnification) independently by two pathologists who were blind to the clinical information with NanoZoomer-XR (Hamamatsu) and Image Pro plus 6.0 digitally. The cut-off values of CD8^+^ T cells were determined by the median value in each study cohort. In TCGA cohort, IMvigor210 cohort and NCT03179943 cohort, the cut-off points were 0.910, 1.035 and 0.530, respectively, while the cut-off value of was set at 25 cells/HPF in ZSHS cohort.

### Classification of molecular subtype

The molecular subtype of each patient was identified through ‘BLCAsubtyping’ R package from https://github.com/cit-bioinfo/BLCAsubtyping in TCGA, IMvigor210 and NCT03179943 cohorts. In ZSHS cohort, we performed IHC single staining of GATA3 and KRT5/6 to classify patients into either Luminal or Basal subtype according to an IHC subtyping method reported in previous articles [25, 26]. GATA3 is predominantly expressed in the Luminal subtype, whereas KRT5/6 is regarded as a Basal marker. IHC analysis determined whether KRT5/6 and GATA3 expression was positive or negative, with the cut-off value being 20% tumor tissue positivity under a 200 × high power field. KRT5/6^+^GATA3^−^ tumors were classified as Basal subtype, while the rest were classified as Luminal subtype.

### Statistical analysis

Kaplan–Meier analysis and log-rank test were performed to conduct survival analyses. Univariate analysis was conducted using the Cox proportional hazards regression model. Pearson's Chi-square test and Fisher’s exact test were applied to detect categorical variables. Statistical *P* values were computed using Kruskal–Wallis test, and detailed statistical tests were described in corresponding figure legends. A *P* value less than 0.05 was considered statistically significant. All statistical analyses were conducted using IBM SPSS Statistics 26.0 and R software 4.1.2.

## Results

### Basal-CD8^+^T^low^ predicts inferior overall survival outcomes in MIBC.

**Fig. 1:**
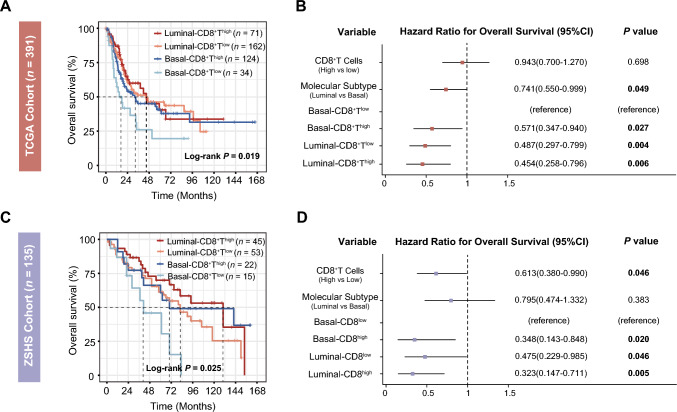
Basal-CD8^+^T^low^predicts inferior overall survival outcomes in MIBC. (**A**) Kaplan–Meier analyses for OS according to four molecular subtype/CD8^+^T cells-stratified subgroups in TCGA cohort. **(B)** Cox regression analyses of OS according to CD8^+^ T cells, molecular subtype and four stratified subgroups in TCGA cohort. **(C)** Kaplan–Meier analyses for OS according to four molecular subtype/CD8^+^T cells-stratified subgroups in ZSHS cohort. **(D)** Cox regression analyses of OS according to CD8^+^ T cells, molecular subtype and four stratified subgroups in ZSHS cohort. Log-rank test was conducted for Kaplan–Meier curves. *P* ≤ 0.05 was considered statistical significance. OS, overall survival

We first explored the association between OS and the four molecular subtype/CD8^+^ T cells-stratified subgroups. The results demonstrated that patients classified under the Basal subtype with low CD8^+^ T cells infiltration (median OS: 17.7 months) exhibited the poorest prognosis, in contrast to the more favorable outcomes observed for the Basal-CD8^+^T^high^ (median OS: 32.0 months), Luminal-CD8^+^T^high^ (median OS: 44.9 months), and Luminal-CD8^+^T^low^ (median OS: 44.3 months) subgroups within the TCGA cohort (log-rank *P* = 0.019, Fig. 1a). Importantly, a coherent pattern emerged from the analysis of the ZSHS cohort, consistently indicating that the Basal-CD8^+^T^high^ subgroup was linked to comparatively inferior OS outcomes (log-rank *P* = 0.025, Fig. 1c). Moreover, univariate analyses confirmed the independent correlation between the Basal-CD8^+^T^high^ subgroup and decreased mortality when contrasted against the Basal-CD8^+^T^high^ (HR: 0.571, 95% CI: 0.347–0.940, *P* = 0.027 and HR: 0.348, 95% CI: 0.143–0.848, *P* = 0.020 in respective cohorts), Luminal-CD8^+^T^low^ (HR: 0.487, 95% CI: 0.297–0.799, *P* = 0.004 and HR: 0.475, 95% CI: 0.229–0.985, *P* = 0.046 in respective cohorts) and Luminal-CD8^+^T^high^ (HR: 0.454, 95% CI: 0.258–0.796, *P* = 0.006 and HR: 0.323, 95% CI: 0.147–0.711, *P* = 0.005 in respective cohorts) subgroups, consistently across both the TCGA and ZSHS cohorts (Fig. 1b, d).

### Basal-CD8^+^T^high^ predicts chemotherapy sensitivity in MIBC.

**Fig. 2:**
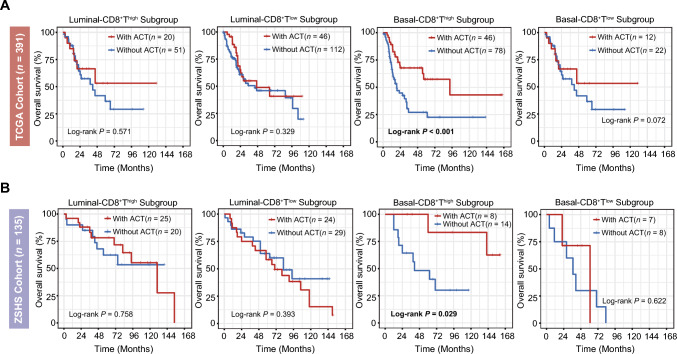
Basal-CD8^**+**^**T**^high^ predicts chemotherapy sensitivity in MIBC. (A) Kaplan–Meier analyses of OS between patients treated with ACT or not in four subgroups stratified by molecular subtype and CD8^+^ T cells in TCGA cohort. **(B)** Kaplan–Meier analyses of OS between patients treated with ACT or not in four subgroups stratified by molecular subtype and CD8^+^T cells in ZSHS cohort. Log-rank test was conducted for Kaplan–Meier curves. *P* ≤ 0.05 was considered statistical significance. OS, overall survival; ACT, adjuvant chemotherapy

The potential of adjuvant chemotherapy (ACT) to extend OS among patients with the Basal subtype was observed (Supplementary Fig. 2A). To delve deeper into the interplay between molecular subtype, CD8^+^ T cells infiltration, and the effectiveness of ACT in MIBC, we conducted further exploration. Our analysis unveiled a noteworthy pattern that only patients characterized by the Basal subtype and high levels of CD8^+^ T cells infiltration experienced discernible clinical advantages from platinum-based chemotherapy. This pattern was consistent across both the TCGA cohort (HR: 0.318, 95% CI: 0.170–0.594, log-rank *P* < 0.001, Fig. 2a), as well as the ZSHS cohort (HR: 0.139, 95% CI: 0.018–1.105, log-rank *P* = 0.029, Fig. 2b).

### Luminal-CD8^+^T^high^ predicts clinical benefits to PD-L1 blockade in MIBC.

We further explored the therapeutic implications for immune checkpoint blockade (ICB) within this paradigm. The Luminal subtype indicated a pronounced survival advantage derived from PD-L1 blockade therapy (log-rank *P* = 0.028), while the Basal subtype did not show similar benefits (log-rank *P* = 0.200, Fig. 2a and Supplementary Fig. 2B). Our exploration further delved into the intricate connection between the four molecular subtype/CD8^+^ T cells-stratified subgroups and their responsiveness to immunotherapy. Utilizing data from the IMvigor210 cohort, our findings highlighted a distinct pattern that solely patients featured by Luminal-CD8^+^T^high^ exhibited significantly enhanced OS (log-rank *P* = 0.009, Fig. 2b) and a heightened objective response rate (ORR, defined as the proportion of patients with complete response and partial response; *P* = 0.002, Fig. 2c) subsequent to treatment with atezolizumab.

**Fig. 3:**
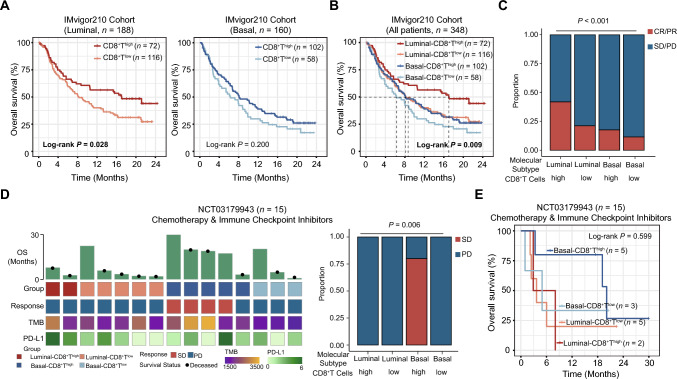
Luminal-CD8^+^T^high^ predicts clinical benefits to PD-L1 blockade in MIBC. (A) Kaplan–Meier analyses for OS of Luminal patients (left panel) and Basal patients (right panel) between high/low CD8^+^ T cells groups after treatment by atezolizumab in IMvigor210 cohort. **(B)** Kaplan–Meier analyses for OS according to four molecular subtype/CD8^+^T cells-stratified subgroups in IMvigor210 cohort. **(C)** Fractions of objective response to atezolizumab between four molecular subtype/CD8^+^T cells-stratified subgroups. **(D)** Characteristics of the response to guadecitabine plus atezolizumab according to four molecular subtype/CD8^+^T cells-stratified subgroups in NCT03179943 cohort. **(E)** Kaplan–Meier analyses of OS on the basis of four molecular subtype/CD8^+^T cells-stratified subgroups patients treated with guadecitabine plus atezolizumab from NCT03179943. Log-rank test was conducted for Kaplan–Meier curves. Kruskal–Wallis test and Chi-square test were applied. *P* ≤ 0.05 was considered statistical significance. OS, overall survival

We next investigated data from the NCT03179943 phase II clinical trial, encompassing 15 patients subjected to guadecitabine in combination with atezolizumab. Specifically, among these patients, those falling under the Basal-CD8^+^T^high^ subgroup (median OS: 20.1 months) exhibited a noteworthy extension in OS through immuno-chemotherapy in comparison with counterparts in the Basal-CD8^+^T^low^ (median OS: 7.1 months), Luminal-CD8^+^T^high^ (median OS: 2.9 months), and Luminal-CD8^+^T^low^ (median OS: 3.7 months, Fig. 3e) subgroups. Importantly, it is worth noting that all individuals who achieved stable disease (SD) belonged to the Basal-CD8^+^T^high^ subgroup (Fig. 3d).

### Immunogenomic features stratified by molecular subtype and CD8^+^ T cells Infiltration.

**Fig. 4:**
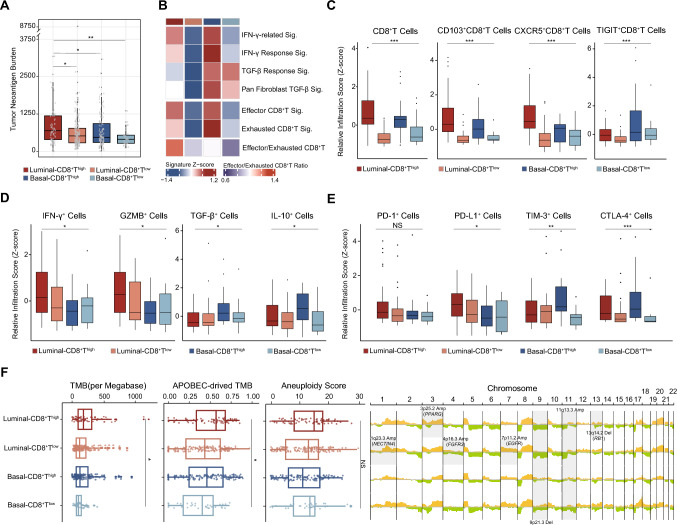
Immunogenomic features stratified by molecular subtype and CD8^+^T cells Infiltration. (A) Evaluation of tumor neoantigen burden between four molecular subtype/CD8^+^T cells-stratified subgroups in TCGA cohort. **(B)** Heatmap illustrating immune functional signatures and immune cells signatures of TCGA cohort to detect the correlation between four molecular subtype/CD8^+^T cells-stratified subgroups and immune microenvironment. **(C)** Correlation between CD8^+^ T cells infiltration, CD103^+^CD8^+^T cells infiltration, CXCR5^+^CD8^+^T cells infiltration, TIGIT^+^CD8^+^T cells infiltration and four molecular subtype/CD8^+^T cells-stratified subgroups in ZSHS. **(D)** Correlation between IFNγ^+^ cells infiltration, GZMB^+^ cells infiltration, TGF-β^+^ cells infiltration, IL10^+^ cells infiltration and four molecular subtype/CD8^+^T cells-stratified subgroups in ZSHS. cohort. **(E)** Correlation between PD-1^+^ cells infiltration, PD-L1^+^ cells infiltration, TIM-3^+^ cells infiltration, CTLA-4^+^ cells infiltration and four molecular subtype/CD8^+^T cells-stratified subgroups in ZSHS. cohort. **(F)** Association between tumor mutational signatures and four molecular subtype/CD8^+^T cells-stratified subgroups in TCGA cohort. Kruskal–Wallis test and Chi-square test were applied.* P* ≤ 0.05 was considered statistical significance

In an effort to unravel the underlying rationale behind the predictive significance of integrating molecular subtype and CD8^+^ T cells infiltration, we next characterized the immune contexture within the context of the four subgroups. As illustrated, patients belonging to the Luminal-CD8^+^T^high^ subgroup exhibited significantly elevated tumor neoantigen burden across both the TCGA and IMvigor210 cohorts (Fig. 4a and Supplementary Fig. 3B). Furthermore, the subgroup characterized by the Luminal subtype and high CD8^+^ T cells infiltration demonstrated notably heightened infiltration of effector CD8^+^ T cells relative to exhausted CD8^+^ T cells (Fig. 4b). To corroborate these outcomes, we further investigated the presence of CD103^+^CD8^+^ T cells and CXCR5^+^CD8^+^ T cells, which demonstrated heightened levels among patients with Luminal-CD8^+^T^high^ subtype. In contrast, the Basal-CD8^+^T^high^ subgroup within the ZSHS cohort exhibited a notable enrichment of TIGIT^+^CD8^+^ T cells (as illustrated in Fig. 4c).

Additionally, patients featured by Luminal-CD8^+^T^high^ exhibited elevated IFN-γ-related and IFN-γ response signatures, while demonstrating reduced TGF-β response and pan fibroblast TGF-β signatures (Fig. 4b) in the TCGA cohort. These findings were replicated in the ZSHS cohort. Patients categorized as Luminal-CD8^+^T^high^ exhibited the highest infiltration of IFN-γ^+^ cells and GZMB^+^ cells. Conversely, TGF-β^+^ cells and IL-10^+^ cells were more abundant within the Basal-CD8^+^T^high^ subgroup (Fig. 4d). Immunohistochemistry analysis further validated these trends. Notably, the Luminal-CD8^+^T^high^ subgroup within the ZSHS cohort displayed an increased presence of various immune checkpoints, including PD-1^+^ cells and PD-L1^+^ cells. Conversely, patients in the Basal-CD8^+^T^high^ subgroup showcased elevated expression of exhaustion markers such as TIM-3^+^ cells and CTLA-4^+^ cells (Fig. 4e).

Through analyses of the TCGA cohort, we found that as the TMB increased, the Luminal-CD8^+^T^high^ subgroup exhibited an augmentation in both APOBEC mutational signatures and aneuploidy scores (Fig. 4f). Furthermore, beyond the prevalent chromosome 9p21.3 deletion and 11q13.3 amplification characteristic of bladder cancer, we observed unique alterations. Specifically, the Luminal-CD8^+^T^high^ subgroup displayed 3p25.2 *PPARG* amplification and 13q14.2 *RB1* deletion, while the Luminal-CD8^+^T^low^ subgroup exhibited 1q23.3 *NECTIN4* amplification, 4p16.3 *FGFR3* amplification, and 7p11.2 *EGFR* amplification (Fig. 4f).

### Luminal-CD8^+^T^low^ predicts superior response to targeted therapies in MIBC

**Fig. 5:**
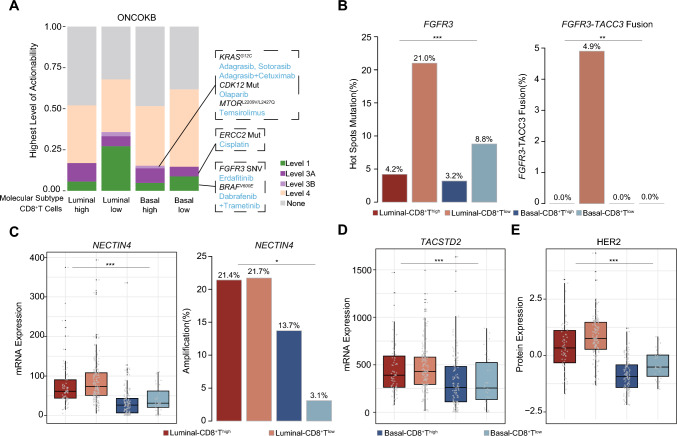
Luminal-CD8^+^**T**^low^ predicts superior response to novel targets treatment in MIBC. (A) Highest levels of therapeutic actionability in MIBC stratified by four molecular subtype/CD8^+^T cells-stratified subgroups. **(B-E)** Distribution of *FGFR3* hotspot mutation, *FGFR3-TACC3* fusion(B) *NECTIN4* mRNA expression, *NECTIN4* amplification(C), *TACSTD2* (Trop-2) mRNA expression(D) and HER2 protein expression among four molecular subtype/CD8^+^T cells-stratified subgroups in TCGA cohort. Kruskal–Wallis test and Chi-square test were applied.* P* ≤ 0.05 was considered statistical significance

In pursuit of further therapeutic avenues for MIBC patients, we sought to establish a connection between emerging biomarkers and the four molecular subtype/CD8^+^T cells-stratified subgroups. Specifically, patients featured by Luminal-CD8^+^T^low^ exhibited a propensity to possess FGFR3 single nucleotide variants (SNVs), which were classified under level 1 of actionability according to the OncoKB database (Fig. 5a). Moreover, *FGFR3* hotspot mutations and *FGFR3*-*TACC* fusion events were notably enriched in patients characterized by the Luminal subtype and low CD8^+^ T cells infiltration (Fig. 5b). This finding underscored the potential benefits that patients with Luminal-CD8^+^T^low^ subtype could derive from FGFR3 inhibitors.

In terms of the potential benefit of antibody–drug conjugate (ADC) drugs, we identified that patients featured by Luminal-CD8^+^T^low^ displayed elevated NECTIN4 mRNA expression and a greater proportion of *NECTIN4* amplifications (Fig. 5c). These observations proposed a potential clinical advantage for patients with Luminal-CD8^+^T^low^ subtype through the utilization of NECTIN4-ADC drugs. Moreover, we ascertained that TACSTD2 mRNA expression was prominently enriched in the Luminal-CD8^+^T^low^ subgroup (Fig. 5d). This finding indicated that patients within this subgroup might benefit from TROP2-ADC drug treatments. Furthermore, the high expression of HER2 protein within the Luminal-CD8^+^T^low^ subgroup (Fig. 5e) indicated the sensitivity of these patients to HER2-targeted drug interventions as well.

## Discussion

Using independent discovery in distinct datasets, we defined four subtypes of MIBC with differ molecular characteristics, immune infiltration, clinical outcomes and therapeutic strategies based on integrating CD8^+^ T cells and molecular subtype (Supplementary Figs. 3 and 4).

Patients belonging to the Luminal-CD8^+^T^high^ subgroup exhibited the most notable levels of TMB and tumor neoantigen burden (TNB). It is well-established that MIBC patients with elevated TMB tend to experience more favorable clinical outcomes when treated with ICB [8, 27]. Remarkably, patients featured by Luminal-CD8^+^T^high^ displayed a large amount of effector CD8^+^T cells infiltration, particularly CD103^+^CD8^+^T cells and CXCR5^+^CD8^+^T cells. Previous studies have consistently demonstrated that the enrichment of CD103^+^CD8^+^T cells and CXCR5^+^CD8^+^T cells is associated with robust cytotoxic activity [14, 16]. This highlights the potential of these infiltrating immune cells to mount an effective defense against tumor cells. Furthermore, research has indicated that the accumulation of APOBEC-mediated mutagenesis signatures serves as a positive prognostic indicator and predictor of favorable response to immunotherapy in bladder cancer patients [28]. In our study, we confirmed that Luminal-CD8^+^T^high^ subgroup exhibited increased levels of both IFN-γ-related signature and APOBEC signature. These findings strongly suggest that patients within this subgroup are highly likely to benefit significantly from immunotherapy interventions. Moreover, patients with Luminal-CD8^+^T^high^ subtype were characterized by the amplification of *PPARG*. Notably, a pre-clinical study demonstrated that the downregulation of *PPARG* expression levels using a PPARγ-inhibitor led to an anti-proliferative effect on tumor cells [29]. Therefore, combining PPARγ-inhibitor therapy with other treatment modalities could potentially provide added value and improved outcomes for patients featured by Luminal-CD8^+^T^high^.

In Luminal-CD8^+^T^low^ subtype of MIBC patients, we observed an enrichment of *FGFR3* mutations and *FGFR3*-*TACC3* fusion. These findings suggest that this particular subtype may be responsive to treatment with FGFR inhibitors. In addition, a promising treatment option is the NECTIN4-ADC enfortumab vedotin (EV), which induced objective clinical responses in 44% of mUC patients with failed treatment for chemotherapy and immunotherapy [30]. We also discovered significantly increased in the mRNA expression of *NECTIN4* and the *NECTIN4* amplification faction within the Luminal-CD8^+^T^low^ subgroup. This suggests that this subgroup is likely to experience clinical benefits from EV treatment. Another ADC, sacituzumab govitecan (SG), targeting TROP2, demonstrated a 27% ORR in mUC patients [31]. In our study, we validated that TACSTD2 mRNA expression is expanded in the Luminal-CD8^+^T^low^ subgroup, suggesting that these patients may also be sensitive to TROP2-ADC. Furthermore, the expression levels of HER2 and EGFR were also elevated in this subgroup, indicating that HER2-ADC and EGFR-targeted drugs might bring clinical benefits to these patients. Currently, bispecific ADCs are undergoing clinical trials and are expected to provide more treatment options for patients in Luminal-CD8^+^T^low^ subgroup.

In Basal-CD8^+^T^high^ subtype of MIBC patients, patients showed a satisfactory median OS after receiving chemotherapy or chemotherapy plus immunotherapy. Although these patients exhibited a remarkable infiltration of CD8^+^ T cells, it is important to note that a significant proportion of these CD8^+^ T cells are characterized dysfunctional TIGIT^+^CD8^+^T cells, which could promote MIBC immune escape [15]. Furthermore, the Basal-CD8^+^T^high^ MIBC subgroup showed an enrichment epithelial-to-mesenchymal transition (EMT) signature, suggesting that Basal-CD8^+^T^high^ tumor cells are more invasive and associated with worse clinical outcomes [32]. Previous studies have shown that TGF-β expression and angiogenesis are associated with resistance to immune checkpoint inhibitors (ICIs) in UC [33], [34]. In our study, we observed that the Basal-CD8^+^T^high^ subgroup displayed the highest TGF-β pathway activity and angiogenesis signature, which may help explain the different clinical outcomes observed in these patients compared to those in the Luminal-CD8^+^T^high^ subgroup [35]. Furthermore, combination therapy with TGF-β inhibitors or anti-angiogenic drugs could provide added value in treating these patients.

Patients with the Basal-CD8^+^T^low^ subtype experienced the worst OS compared to other subtypes. Numerous researches have demonstrated the detrimental role of WNT5A, a protein involved in cell signaling, in driving chemotherapy resistance and promoting the expression of stemness markers in bladder cancer cells [36]. This effect is primarily due to the activation of the WNT/β-catenin signaling pathway, which has been extensively linked to poor prognosis and drug resistance across various cancer types [37–40]. We also found that the activity of the WNT pathway significantly increases in patients with the Basal-CD8^+^T^low^ subtype. CDKs play a crucial role in regulating the cell cycle and their inhibitors have shown promising results in targeting cancer cells, repressing their uncontrolled growth, and potentially sensitizing them to chemotherapy [41]. By specifically targeting the dysregulated cell cycle pathway in patients with Basal-CD8^+^T^low^ tumor, CDK inhibitors have the potential to improve treatment outcomes and enhance OS rates in this challenging patient population [42].

Our research has several limitations. Firstly, given the retrospective and exploratory design of our study, the results still require further validation within the framework of more extensive, multi-centered clinical cohorts. Secondly, while we do present compelling evidence of the correlation between four molecular subtype/CD8^+^T cells-stratified subgroups and the survival benefit from ACT in our local cohort, validating our immunotherapy-related findings still requires further investigation. Thirdly, the patient number of some cohorts included is too small to achieve statistical significance for overall survival which needs further validation. Fourthly, the four subgroups are different in patients’ numbers and heterogeneity, and more validation is needed to enhance the credibility of the study. Finally, we recognize the need for functional research and prospective experiments to validate our hypotheses.

**Fig. 6:**
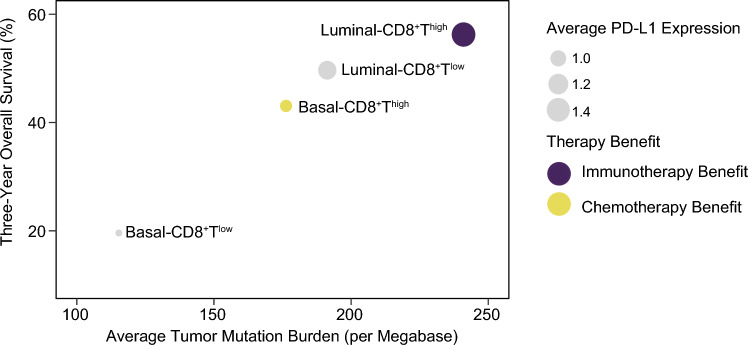
Clinical outcomes summary for patients with MIBC organized by four molecular subtype/CD8^+^T cells-stratified subgroups. Three-year overall survival was calculated using Kaplan–Meier analysis for MIBC patients. The average PD-L1 expression (scaled dot size) and effective treatment strategies (dot color) are represented from multi-cohort analyses

In summary, we integrated findings from our study analyses of patients with MIBC (Fig. 6). Our study highlighted the potential of the immunomolecular subtype based on molecular subtype (Luminal and Basal) and CD8^+^T cells infiltration. For patients featured by Luminal-CD8^+^T^high^, along with the highest level of TMB and PD-L1 expression, these patients were likely to benefit from immunotherapy. Patients with Luminal-CD8^+^T^low^ tumors are more likely to benefit from FGFR3 inhibitors and ADC drugs including NECTIN4-ADC, TROP2-ADC and HER2-ADC. Patients with Basal-CD8^+^T^high^ subtype were sensitive to chemotherapy or chemotherapy plus immunotherapy. Patients featured by Basal-CD8^+^T^low^ had the worst three-year OS.

## Conclusion

Our study defined an immune-molecular subtyping classification based on CD8^+^T cells and molecular subtypes. This classification is hoped to serve as a foundation for developing tailored therapeutic approaches for MIBC patients.

### Supplementary Information

Below is the link to the electronic supplementary material.

Supplementary file1 (DOCX 904 KB)

## Data Availability

Data and materials generated that are relevant to the results are included in this article. Other data are available from the corresponding author Prof. Zhang upon reasonable request.

## Abbreviations

ACT: Adjuvant chemotherapy
ADC: Antibody–drug conjugate
CNV: Copy number variants
CTLA-4: Cytotoxic T lymphocyte-associated antigen-4
EMT: Epithelial-mesenchymal transition
FGFR3: Fibroblast growth factor receptor 3
FPKM: Fragments per kilobase of transcript per million mapped reads
GISTIC: Genomic Identification of significant targets in cancer
GZMB: Granzyme B
HER2: Human epithelial growth factor receptor-2
ICB: Immune checkpoint blockade
ICIs: Immune checkpoint inhibitors
IFN-γ: Interferon-γ
IHC: Immunohistochemistry
IL-10: Interleukin 10
MIBC: Muscle-invasive bladder cancer
mUC: Metastatic urothelial carcinoma
NMIBC: Non-muscle-invasive bladder cancer
ORR: Objective response rate
OS: Overall survival
PD-1: Programmed cell death 1
PD-L1: Programmed cell death-ligand 1
SD: Stable disease
ssGSEA: Single sample gene set enrichment analysis
TCGA: The Cancer Genome Atlas
TGF-β: Transforming growth factor-β
TIM-3: T cell immunoglobulin domain and mucin domain-3
TMA: Tissue microarray
TMB: Tumor mutation burden
TNB: Tumor neoantigen burden
ZSHS: Zhongshan hospital
