# Supplementary material for: Integrating molecular subtype and CD8+ T cells infiltration to predict treatment response and survival in muscle-invasive bladder cancer
Source: Cancer Immunol Immunother. 2024 Mar 2;73(4):66. doi: 10.1007/s00262-024-03651-3 (PMC10908619; doi:10.1007/s00262-024-03651-3)
Supplement: Supplementary file 1 — Supplementary file1 (DOCX 904 KB) [file 262_2024_3651_MOESM1_ESM.docx]

**Integrating Molecular Subtype and CD8^+^ T Cells Infiltration to Depict the Clinical Outcomes and Therapeutic Strategies in Muscle-Invasive Bladder Cancer**

**Supplementary Data**

Supplementary Figure 1. Flow chart of cohort selection and representative Images of CD8^+^T cells.

Supplementary Figure 2. Correlation of molecular subtype and CD8^+^T cells infiltration with clinical outcomes in patients treated with chemotherapy or immunotherapy.

Supplementary Figure 3. Pathway activity across subgroups based on CD8^+^ T cells infiltration and molecular subtype in MIBC.

Supplementary Figure 4. Overview of features and potential therapeutic implications per subtype for MIBC.

Supplementary Table 1. Clinicopathological characteristics in TCGA and ZSHS cohorts.

Supplementary Table 2. Clinicopathological characteristics in IMvigor210 cohort.

Supplementary Table 3. Clinicopathological characteristics in NCT03179943 cohort.

Supplementary Table 4. Gene signature list.

Supplementary Table 5. Immunohistochemistry antibodies list.


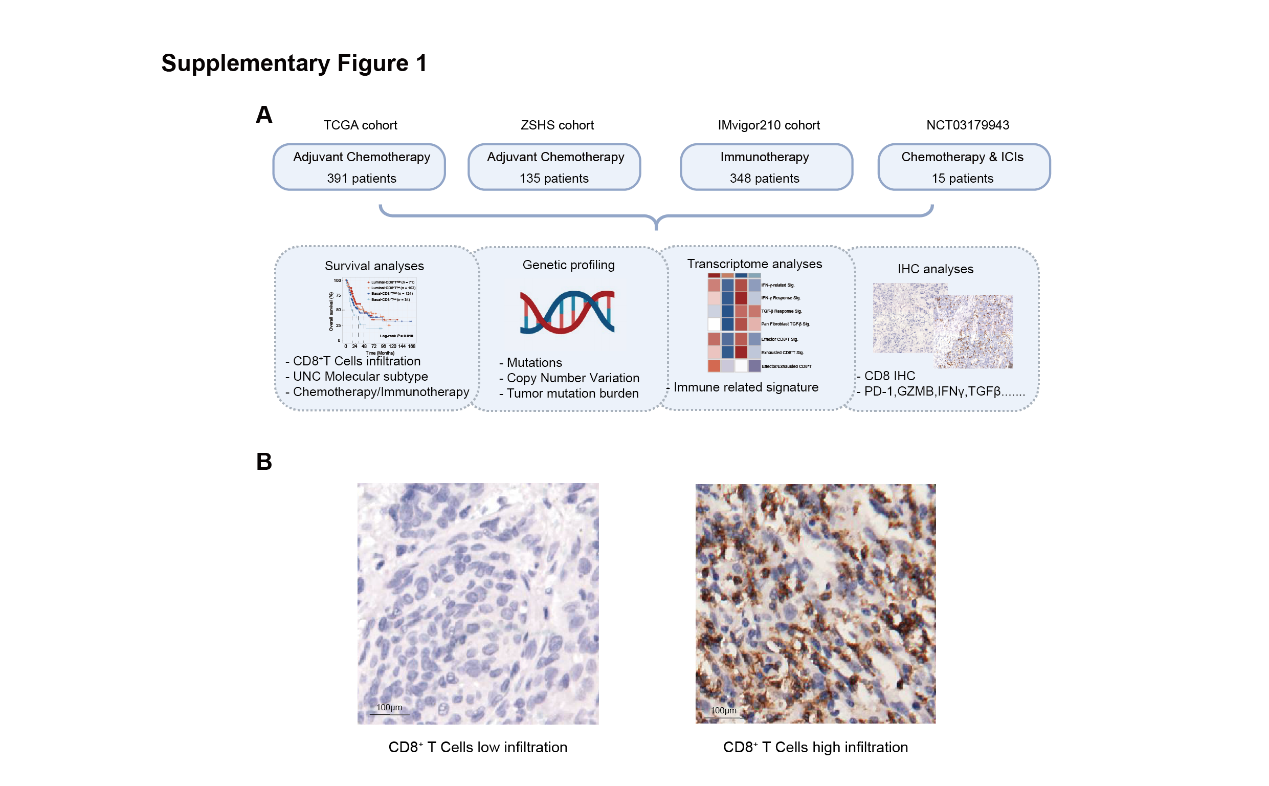


**Supplementary Figure 1. Flow chart of cohort selection and representative Images of CD8^+^T cells. (A)** Comprehensive information about patients included in this study. **(B)** Immunohistochemistry staining showed CD8^+^T cells infiltration in MIBC.


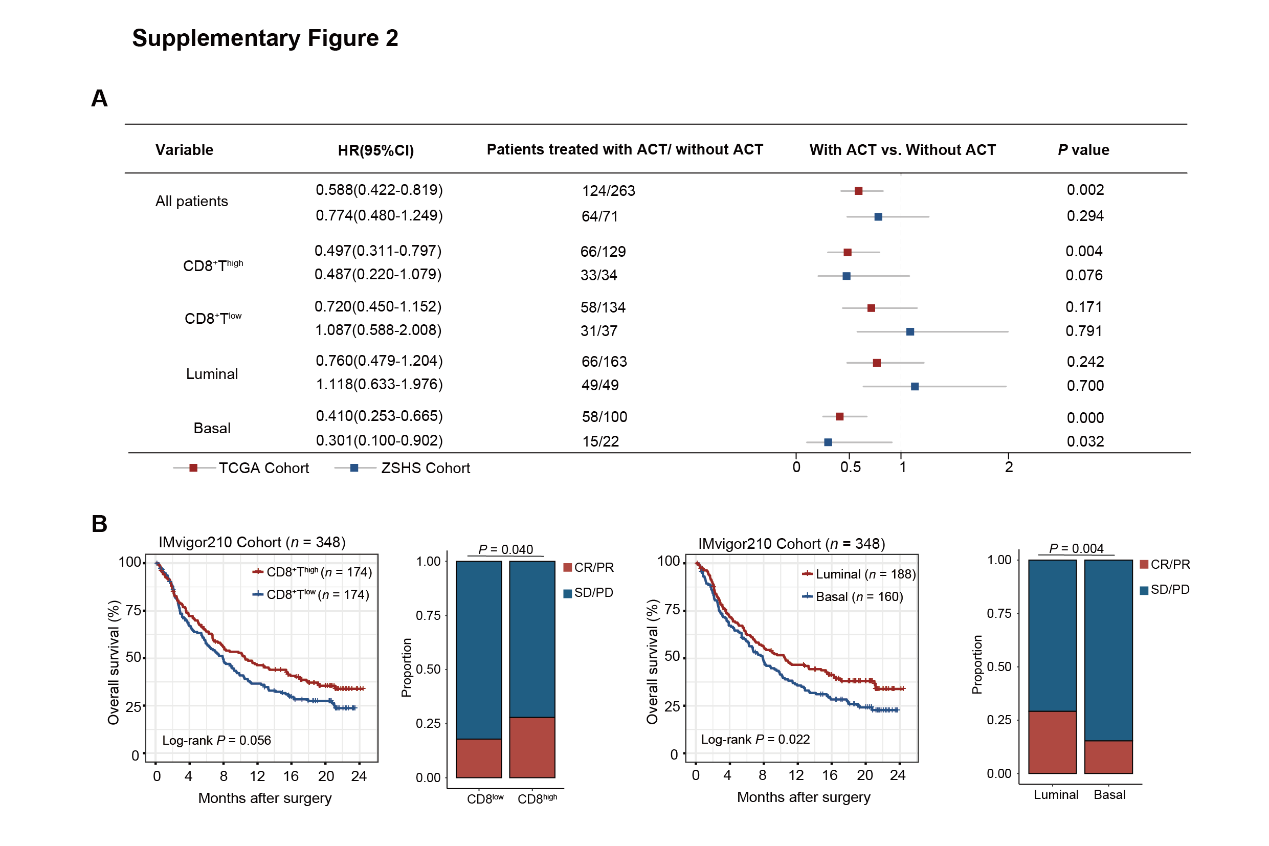


**Supplementary Figure 2. Correlation of molecular subtype and CD8^+^T cells infiltration with clinical outcomes in patients treated with chemotherapy or immunotherapy. (A)** Cox regression analyses of OS in patients with or without ACT according to CD8^+^ T cells infiltration and molecular subtype in TCGA cohort (red) and ZSHS cohort (blue). **(B)** Kaplan-Meier analyses of OS in IMvigor210 cohort according to CD8^+^ T cells infiltration (left) and molecular subtype (right) in IMvigor210 cohort. Stacked bar displaying the fractions of objective response to atezolizumab between CD8^+^ T cells infiltration (left) and molecular subtype (right). Log-rank test was conducted for Kaplan–Meier curves. Data were analyzed by Pearson’s chi-square test. *P* ≤ 0.05 was considered statistical significance. OS, overall survival; ACT, adjuvant chemotherapy.


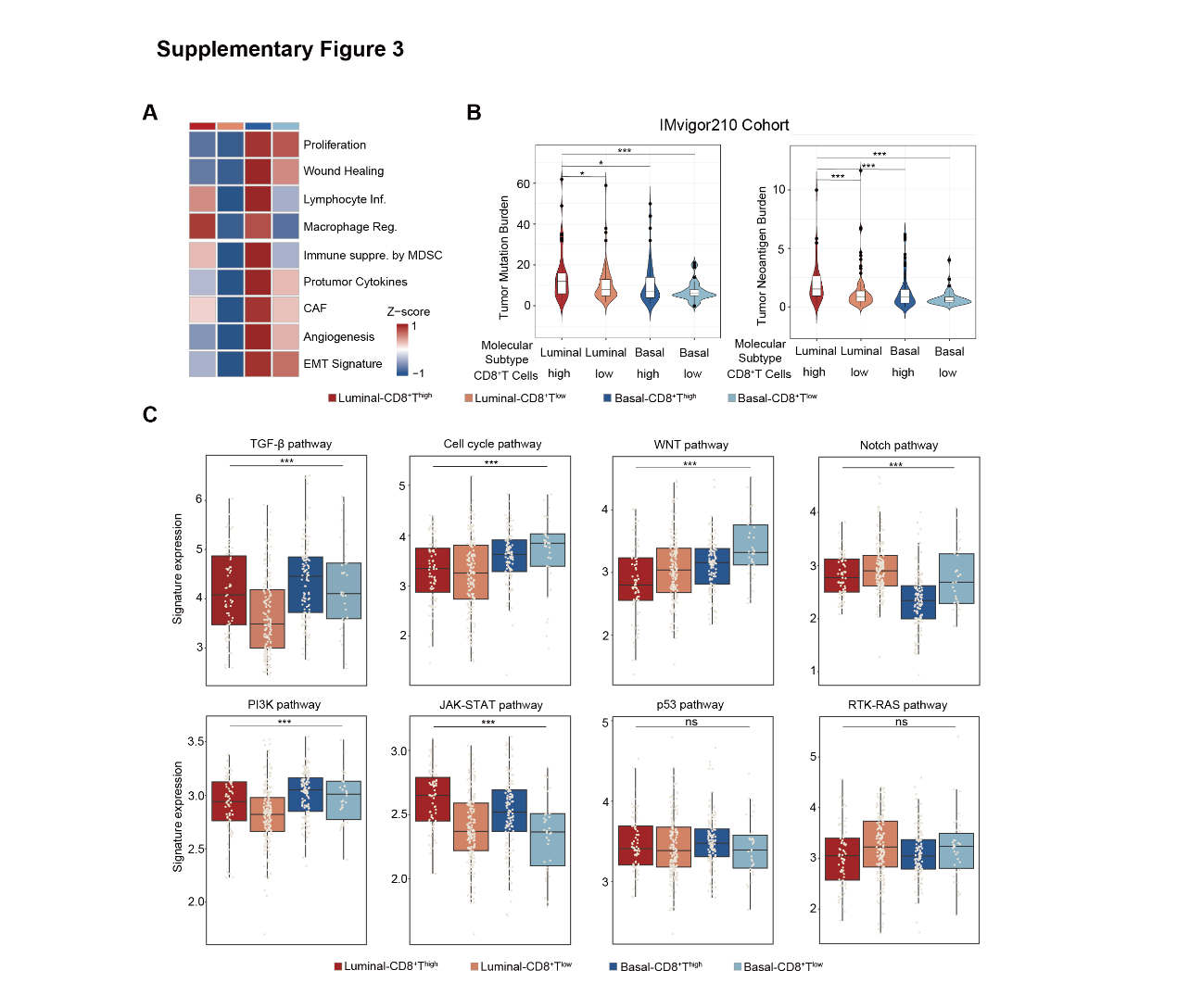


**Supplementary Figure 3. Pathway activity across subgroups based on CD8^+^ T cells infiltration and molecular subtype in MIBC. (A)** Correlation between tumor microenvironment related signatures and four molecular subtype/CD8^+^T cells-stratified subgroups in TCGA cohort. **(B)** Association between TMB/TNB and four molecular subtype/CD8^+^T cells-stratified subgroups in IMvigor210 cohort. **(C)** Association between oncogenic pathway activity and four molecular subtype/CD8^+^T cells-stratified subgroups in TCGA cohort. Kruskal-Wallis test and Chi-square test were applied. *P* ≤ 0.05 was considered statistical significance.


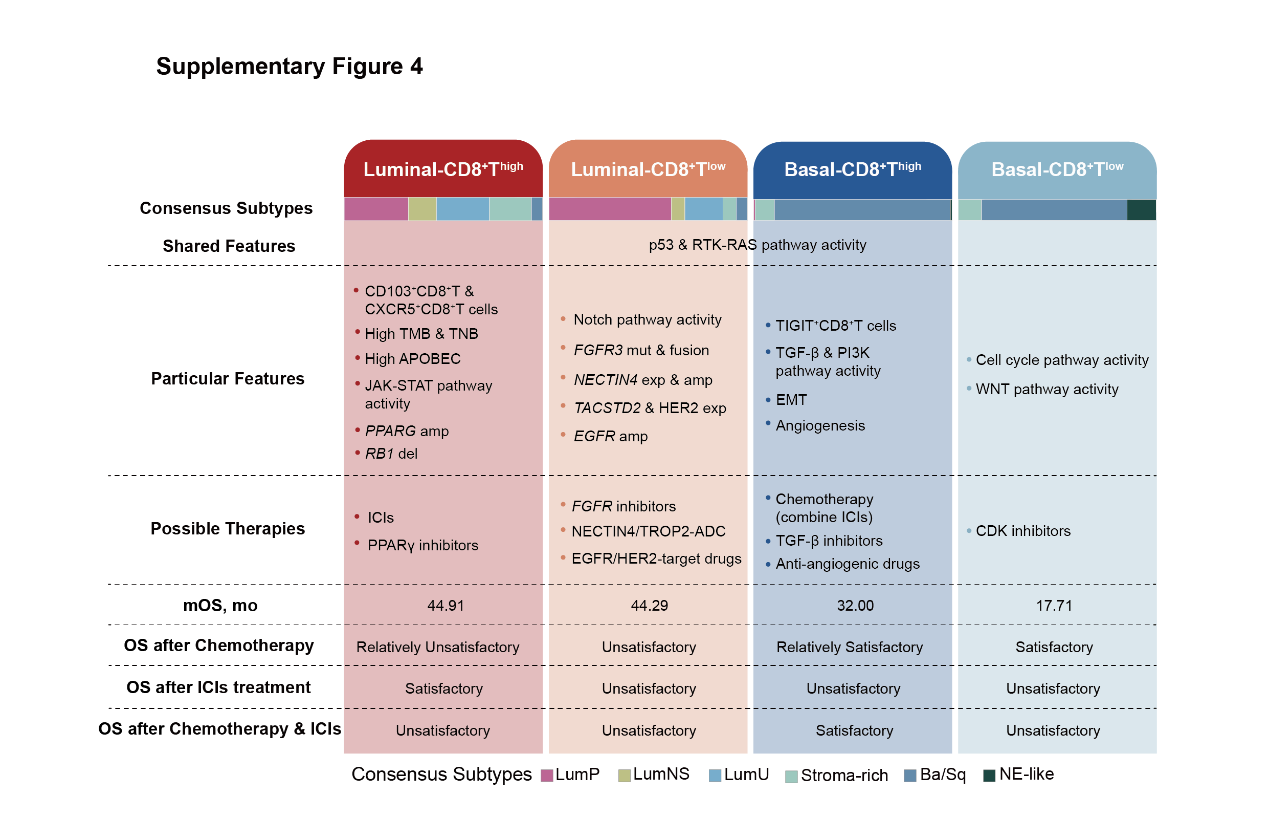


**Supplementary Figure 4. Overview of features and potential therapeutic implications per subtype for MIBC.** TMB, tumor mutational burden; TNB, tumor neoantigen burden; ICIs, immune checkpoint inhibitions; EMT, epithelial-to-mesenchymal transition.

| **Supplementary Table 1. Clinicopathological characteristics in TCGA and ZSHS cohorts.** | | | | | | | | | | | | | |
| --- | --- | --- | --- | --- | --- | --- | --- | --- | --- | --- | --- | --- | --- |
| **Characteristics** | **TCGA cohort (*n* = 391)** | | | | | |  | **ZSHS cohort (*n* = 135)** | | | | | |
|  | No. | Luminal-  CD8^+^T^high^ | Luminal-  CD8^+^T^low^ | Basal-  CD8^+^T^high^ | Basal-  CD8^+^T^low^ | ***P*** |  | **No.** | Luminal-  CD8^+^T^high^ | Luminal-  CD8^+^T^low^ | Basal-  CD8^+^T^high^ | Basal-  CD8^+^T^low^ | ***P*** |
| **Total** | 391 | 71 | 162 | 124 | 34 |  |  | 135 | 45 | 53 | 22 | 15 |  |
| **Age** |  |  |  |  |  | 0.340 |  |  |  |  |  |  | 0.430 |
| <60 | 84 | 14 | 42 | 22 | 6 |  |  | 50 | 15 | 24 | 7 | 4 |  |
| ≥60 | 307 | 57 | 120 | 102 | 28 |  |  | 85 | 30 | 29 | 15 | 11 |  |
| **Gender** |  |  |  |  |  | 0.474 |  |  |  |  |  |  | 0.724 |
| Male | 285 | 54 | 122 | 84 | 25 |  |  | 112 | 37 | 45 | 19 | 11 |  |
| Female | 106 | 17 | 40 | 40 | 9 |  |  | 23 | 8 | 8 | 3 | 4 |  |
| **AJCC stage** |  |  |  |  |  | **0.027** |  |  |  |  |  |  | 0.565 |
| II | 125 | 26 | 60 | 33 | 6 |  |  | 82 | 30 | 29 | 14 | 9 |  |
| III | 137 | 18 | 50 | 56 | 13 |  |  | 45 | 12 | 21 | 8 | 4 |  |
| IV | 129 | 27 | 52 | 35 | 15 |  |  | 8 | 3 | 3 | 0 | 2 |  |
| **pT stage** |  |  |  |  |  | 0.229 |  |  |  |  |  |  | 0.593 |
| pT2 | 113 | 22 | 56 | 29 | 6 |  |  | 85 | 32 | 29 | 14 | 10 |  |
| pT3 | 189 | 30 | 71 | 71 | 17 |  |  | 29 | 6 | 16 | 4 | 3 |  |
| pT4 | 56 | 11 | 22 | 17 | 6 |  |  | 21 | 7 | 8 | 4 | 2 |  |
| **pN stage** |  |  |  |  |  | 0.163 |  |  |  |  |  |  | 0.405 |
| pN0 | 228 | 34 | 99 | 78 | 17 |  |  | 127 | 42 | 50 | 22 | 13 |  |
| pN1+ | 123 | 27 | 48 | 34 | 14 |  |  | 8 | 3 | 3 | 0 | 2 |  |
| **Grade** |  |  |  |  |  | **<0.001** |  |  |  |  |  |  | 0.057 |
| Low | 20 | 0 | 19 | 1 | 0 |  |  | 22 | 6 | 14 | 1 | 1 |  |
| High | 369 | 71 | 142 | 122 | 34 |  |  | 113 | 39 | 39 | 21 | 14 |  |
| **LVI** |  |  |  |  |  | 0.533 |  |  |  |  |  |  | 0.953 |
| Absent | 125 | 18 | 55 | 41 | 11 |  |  | 49 | 17 | 20 | 7 | 5 |  |
| Present | 142 | 30 | 57 | 45 | 10 |  |  | 86 | 28 | 33 | 15 | 10 |  |
| **ACT** |  |  |  |  |  | 0.435 |  |  |  |  |  |  | 0.499 |
| Applied | 124 | 20 | 46 | 46 | 12 |  |  | 64 | 25 | 24 | 8 | 7 |  |
| Not applied | 263 | 51 | 112 | 78 | 22 |  |  | 71 | 20 | 29 | 14 | 8 |  |
| Abbreviations: AJCC: American Joint Committee on Cancer; LVI: lymphatic vessel invasions; ACT: adjuvant chemotherapy. | | | | | | | | | | | | | |
| **P* value was used from Pearson’s chi-square test, significant *P* value < 0.05 was shown in bold. | | | | | | | | | | | | | |

| **Supplementary Table 2. Clinicopathological characteristics in IMvigor210 cohort.** | | | | | | |
| --- | --- | --- | --- | --- | --- | --- |
| **Characteristics** | **IMvigor210 cohort (*n* = 348)** | | | | | |
|  | No. | Luminal-CD8^+^T^high^ | Luminal-CD8^+^T^low^ | Basal-CD8^+^T^high^ | Basal-CD8^+^T^low^ | ***P*** |
| **Total** | 348 | 72 | 116 | 102 | 58 |  |
| **Gender** |  |  |  |  |  | 0.633 |
| Male | 272 | 58 | 87 | 83 | 44 |  |
| Female | 76 | 14 | 29 | 19 | 14 |  |
| **AJCC stage** |  |  |  |  |  |  |
| IV | 348 | 72 | 116 | 102 | 58 |  |
| **PD-L1^+^IC Level** |  |  |  |  |  | **<0.001** |
| IC0 | 97 | 7 | 59 | 9 | 22 |  |
| IC1 | 132 | 33 | 45 | 29 | 25 |  |
| IC2+ | 118 | 32 | 12 | 63 | 11 |  |
| **TMB** |  |  |  |  |  | **0.006** |
| Low (<10) | 160 | 24 | 58 | 47 | 31 |  |
| High (≥10) | 112 | 33 | 41 | 29 | 9 |  |
| **Response** |  |  |  |  |  | **<0.001** |
| CR | 25 | 13 | 2 | 7 | 3 |  |
| PR | 43 | 13 | 19 | 8 | 3 |  |
| SD | 63 | 9 | 18 | 28 | 8 |  |
| PD | 167 | 27 | 60 | 42 | 38 |  |
| **Event** |  |  |  |  |  | **0.006** |
| Death | 232 | 37 | 77 | 72 | 46 |  |
| Abbreviations: TMB: tumor mutation burden; PD-L1^+^IC: PD-L1 expression on immune cells; CR: Complete Response; PR: Partial Response; SD: Stable Disease; PD: Progressive Disease | | | | | | |
| **P* value was used from Pearson’s chi-square test, significant *P* value < 0.05 was shown in bold. | | | | | | |

| **Supplementary Table 3. Clinicopathological characteristics in NCT03179943 cohort.** | | | | | | |
| --- | --- | --- | --- | --- | --- | --- |
| **Characteristics** | **NCT03179943 cohort (*n* = 15)** | | | | | |
|  | No. | Luminal-CD8^+^T^high^ | Luminal-CD8^+^T^low^ | Basal-CD8^+^T^high^ | Basal-CD8^+^T^low^ | ***P*** |
| **Total** | 15 | 2 | 5 | 5 | 3 |  |
| **Age** |  |  |  |  |  | 0.444 |
| <60 | 8 | 0 | 3 | 3 | 2 |  |
| ≥60 | 7 | 2 | 2 | 2 | 1 |  |
| **Gender** |  |  |  |  |  | 0.120 |
| Male | 12 | 2 | 4 | 5 | 1 |  |
| Female | 3 | 0 | 1 | 0 | 2 |  |
| **AJCC stage** |  |  |  |  |  |  |
| IV | 15 | 2 | 5 | 5 | 3 |  |
| **Response** |  |  |  |  |  | **0.012** |
| CR | 0 | 0 | 0 | 0 | 0 |  |
| PR | 0 | 0 | 0 | 0 | 0 |  |
| SD | 4 | 0 | 0 | 4 | 0 |  |
| PD | 11 | 2 | 5 | 1 | 3 |  |
| **Event** |  |  |  |  |  | 0.714 |
| Death | 11 | 2 | 4 | 3 | 2 |  |
| Abbreviations: CR: Complete Response; PR: Partial Response; SD: Stable Disease; PD: Progressive Disease | | | | | | |
| **P* value was used from Pearson’s chi-square test; significant *P* value < 0.05 was shown in bold. | | | | | | |

| **Supplementary Table 4. Gene signature list.** | | |
| --- | --- | --- |
| **Signature** | **Genes** | **Source** |
| CD8^+^ T cells | *CD8A, CD8B, EOMES, FASLG, GNLY, GZMA, GZMB, GZMK, IFNG, PRF1, TBX21, ZAP70* | PMID: 34019806 |
| IFN-γ-related | *CD8A, CCL5, CD27, CD274, PDCD1LG2, CD276, CMKLR1, CXCL9, CXCR6, HLA-DQA1, HLA-DRB1, HLA-E, IDO1, LAG3, NKG7, PSMB10, STAT1, TIGIT* | PMID:28650338 |
| Pan Fibroblast TGF-β | *ACTA2, ACTG2, ADAM12, ADAM19 , CNN1, COL4A1, CTGF, CTPS1, FAM101B, FSTL3, HSPB1, IGFBP3, PXDC1, SEMA7A, SH3PXD2A, TAGLN, TGFBI, TNS1, TPM1* | PMID: 29443960 |
| Effector CD8^+^T | *CD8A, CXCL10, CXCL9, GZMA, GZMB, IFNG, PRF1, TBX21* | PMID: 26952546 |
| Exhausted CD8^+^T | *Tnfsf6, Pbx3, Gp49b, Cd244, Ccl3, Eomes, Casp3, Plscr1, Kdt1, Ctla4, Pdcd1, Ler5, Rgs16, A430109M19Ri, Tnfrsf9, Penk1, Eomes, Coch, Ptpn13, Tcrg-v4, Nr4a2, Cd160, Ptger4, Ccl4, Wbp5, Gpr56, 1110067D22Rik, Entpd1, Sh2d2a, 45173, Lsg20, Trim47, Serpina3g, Casp4, 9130009C22Rik, C79248, Lag3, Nr4a2, Nfatc1, Car2, C330007P06Rik, Gas2, Mx1, 4631408O11Rik, GPD2, 2700084L22RIK, RNF11, CAPZB, TUBB2, BUB1, JAK3, 9130410M22RIK, CD9, TCRG-V4, 1810054d07RIK, RCN, 2010100O12RIK, SYB1, ETF1, CPA3, CD7, ART3, 1810035L17RIK, ATF1, PRKWNK1, MTV43, CIT, CCRL2, ADFP, D8ERTD531E, TCEA2, MYH4, TNFRSF1A, SPP1, S100A13, PON2, AI181996, G1P2, TANK, SHKBP1, 2510004L01RIK, D15ERTD781E, LCSBP1, BC024955, GDF3, ITGAV, 1110006I15RIK, CPSF2, KIK6, CPT2, LMAN2, TOR3A* | PMID: 17950003 |
| Signature score was calculated by the single sample gene set enrichment analysis (ssGSEA) algorithms. | | |

| **Supplementary Table 5. Immunohistochemistry antibodies list.** | | | | | |
| --- | --- | --- | --- | --- | --- |
| **IHC** | **Antibody** | **Clonality Species** | **Company** | **Product No.** | **Diluted** |
| CD8^+^ T cells | Anti-CD8 alpha antibody | Monoclonal Mouse Anti-human | Abcam | ab17147 | 1:100 |
| GATA3^+^ cells | Anti-GATA3 antibody | Monoclonal Rabbit Anti-human | Abcam | ab199428 | 1:100 |
| KRT5/6^+^ cells | Anti-Cytokeratin 5+6 antibody | Monoclonal Mouse Anti-human | Abcam | ab17133 | 1:25 |
| CD103^+^CD8^+^ T cells | Anti-CD103 antibody | Monoclonal Rabbit Anti-human | Abcam | ab129202 | 1:300 |
|  | Anti-CD8 alpha antibody | Monoclonal Mouse Anti-human | Abcam | ab17147 | 1:100 |
| CXCR5^+^CD8^+^ T cells | Anti- CXCR5 antibody | Polyclonal Rabbit Anti-human | Abcam | ab46218 | 1:1000 |
|  | Anti-CD8 alpha antibody | Monoclonal Mouse Anti-human | Abcam | ab17147 | 1:100 |
| TIGIT^+^CD8^+^ T cells | Anti-TIGIT antibody | Monoclonal Rabbit Anti-human | Abcam | ab243903 | 1:50 |
|  | Anti-CD8 alpha antibody | Monoclonal Mouse Anti-human | Abcam | ab17147 | 1:100 |
| IFN-γ^+^ cells | Anti-IFN-γ antibody | Monoclonal Rabbit Anti-human | Abcam | ab9657 | 1:300 |
| GZMB^+^ cells | Anti-GZMB antibody | Monoclonal Rabbit Anti-human | Abcam | ab4059 | 1:200 |
| TGF-β^+^ cells | Anti- TGF-β antibody | Monoclonal Goat Anti-human | RD | AB-246-NA | 1:100 |
| IL-10^+^ cells | Anti-PD-L1 antibody | Monoclonal Mouse Anti-human | Abcam | ab134742 | 1:200 |
| PD-1^+^ cells | Anti-PD1 antibody | Monoclonal Mouse Anti-human | Abcam | ab52587 | 1:1000 |
| PD-L1^+^ cells | Anti-PD-L1 antibody | Monoclonal Rabbit Anti-human | Abcam | ab228415 | 1:500 |
| TIM-3^+^ cells | Anti- TIM-3 antibody | Monoclonal Rabbit Anti-human | Abcam | ab185703 | 1:100 |
| CTLA-4^+^ cells | Anti-CTLA-4 antibody | Monoclonal Mouse Anti-human | Santa-Cruz | sc-376016 | 1:100 |
| Abbreviations: IFN-γ: interferon-γ; GZMB: granzyme B; TGF-β: transforming growth factor-β; IL10: interleukin 10; PD-1: Programmed cell death protein 1; PD-L1: programmed cell death 1 ligand 1; TIM-3: T-cell immunoglobulin mucin-3; CTLA-4: Cytotoxic T lymphocyte-associated protein 4 | | | | | |
